# Myofibre Hyper-Contractility in Horses Expressing the Myosin Heavy Chain Myopathy Mutation, *MYH1^E321G^*

**DOI:** 10.3390/cells10123428

**Published:** 2021-12-06

**Authors:** Julien Ochala, Carrie J. Finno, Stephanie J. Valberg

**Affiliations:** 1 Department of Biomedical Sciences, University of Copenhagen, 2200 Copenhagen, Denmark; 2 Centre of Human and Applied Physiological Sciences, Faculty of Life Sciences & Medicine, School of Basic and Medical Biosciences, King’s College London, London SE1 1UL, UK; 3 Randall Centre for Cell and Molecular Biophysics, Faculty of Life Sciences & Medicine, School of Basic & Medical Biosciences, King’s College London, London SE1 1UL, UK; 4 Department of Population Health and Reproduction, School of Veterinary Medicine, University of California at Davis, Davis, CA 95616, USA; cjfinno@ucdavis.edu; 5 Large Animal Clinical Sciences, College of Veterinary Medicine, Michigan State University, East Lansing, MI 48824, USA; valbergs@msu.edu

**Keywords:** congenital myopathy, inflammation, myosin, *MYH1*, muscle fibre, mechanics

## Abstract

Myosinopathies are defined as a group of muscle disorders characterized by mutations in genes encoding myosin heavy chains. Their exact molecular and cellular mechanisms remain unclear. In the present study, we have focused our attention on a *MYH1*-related E321G amino acid substitution within the head region of the type IIx skeletal myosin heavy chain, associated with clinical signs of atrophy, inflammation and/or profound rhabdomyolysis, known as equine myosin heavy chain myopathy. We performed Mant-ATP chase experiments together with force measurements on isolated IIx myofibres from control horses (*MYH1^E321G−/−^*) and Quarter Horses homozygous (*MYH1^E321G+/+^*) or heterozygous (*MYH1^E321G+/−^*) for the E321G mutation. The single residue replacement did not affect the relaxed conformations of myosin molecules. Nevertheless, it significantly increased its active behaviour as proven by the higher maximal force production and Ca^2+^ sensitivity for *MYH1^E321G+/+^* in comparison with *MYH1^E321G+/−^* and *MYH1^E321G−/−^* horses. Altogether, these findings indicate that, in the presence of the E321G mutation, a molecular and cellular hyper-contractile phenotype occurs which could contribute to the development of the myosin heavy chain myopathy.

## 1. Introduction

Myosinopathies are a heterogeneous group of congenital myopathies clinically ranging from late onset mild muscle dysfunction to early lethal symptomatic manifestations [1,2]. They are associated with mutations in the *MYH3*, *MYH8*, *MYH7*, *MYH2*, *MYH1* and *MYH4* genes, all encoding for myosin heavy chain isoforms present in the foetus, neonatal and/or adult skeletal muscles [1,2]. Despite this knowledge and despite the fact that we know that more than 90% are missense mutations, resulting in the replacement of just one residue in the head domain, converter or tail regions of the myosin molecule [3,4,5,6], the exact pathophysiological mechanisms remain unclear.

The head domain of myosin heavy chains is essential for molecular force production and motion [7]. Hence, subtle amino acid substitutions in this particular region are likely to lead to severe molecular and cellular contractile impairments followed by negative muscle consequences [1,2]. Recently, in Quarter Horses, the most popular breed in the USA with over 4 million horses registered by the American Quarter Horse Association, a pathogenic co-dominant mutation in the *MYH1* gene encoding for the fast skeletal myosin heavy chain type IIx (*MYH1^E321G^*) has been identified in the motor domain and linked to a detrimental inflammatory myopathy known as equine immune-mediated myositis [8,9,10]. The *MYH1^E321G^* mutation is present in 6% of the Quarter Horse breed [9]. Clinically, horses homozygous or heterozygous for *MYH1^E321G^* experience rapid onset of malaise, stiffness, skeletal muscle atrophy and weakness; histopathologically, the affected horses display inflammatory infiltrates within type IIx myofibres, particularly CD^4+^-, CD^8+^-, and CD^20+^-positive lymphocytes [8,11,12]. Of interest, immune-mediated myositis also affects humans and dogs with sub-types including inclusion body myositis, polymyositis, and dermatomyositis, including an autoimmunity to canine masticatory muscle myosin [13,14].

Another *MYH1^E321G^*- related phenotype has also been discovered in young Quarter Horses in which heterozygous and homozygous animals suddenly develop a profound non-exertional rhabdomyolysis notably characterized by muscle stiffness, pain and inability to rise. Additionally, these symptoms are often accompanied with muscle atrophy in homozygous horses [10]. Histochemically, there can be little difference in myosin ATPase staining of type IIa and IIx fibres in 60% of homozygous horses, whereas IIa and IIx fibre types are readily distinguished in immunofluorescent staining using myosin antibodies [10]. The term equine myosin heavy chain myopathy (MYHM) is now used to encompass the two types of phenotypes of *MYH1^E321G^* presented above (equine immune-mediated myositis and profound non-exertional rhabdomyolysis). Hence, unveiling how *MYH1^E321G^* disrupts molecular and cellular contractility may be translatable to the various forms of equine MYHM diseases.

Single amino acid substitutions near *MYH1^E321G^* (*MYH7^R369Q^*, *MYH7^E374V^*, *MYH7^A381D^* and *MYH7^Y386C^*) are associated with deleterious hypertrophic or dilated cardiomyopathies [15]. One of the major triggers of the cardiac muscle phenotypes for these mutations is thought to be a direct destabilisation of the myosin interacting-heads motif [15]. The interacting-heads motif is crucial for myosin head positioning by promoting the super-relaxed state and limiting potential interactions between myosin heads and actin [16]. Hence, in the present study, we initially hypothesized that *MYH1^E321G^* would also impact myosin head positioning and super-relaxed state, and, thus, would alter myofibre force-generating capacity, overall contributing to the development of equine MYHM. To test this hypothesis, we used Mant-ATP chase experiments and force measurements of individual muscle fibres isolated from control Quarter Horses (*MYH1^E321G^*^−/−^) and horses homozygous (*MYH1^E321G^*^+/+^) or heterozygous (*MYH1^E321G^*^+/−^) for the mutation.

## 2. Materials and Methods

### 2.1. Animals

Muscle biopsy specimens were taken from semimembranosus muscles of equine immune-mediated myositis-affected Quarter Horses who were homozygous (*MYH1^E321G^*^+/+^; *n* = 2; one 1-year-old male and one 5-month-old female) or heterozygous (*MYH1^E321G^*^+/−^; *n* = 2; one 1-year-old male and one 2-year-old female) for the *MYH1* mutation. All samples were shipped on ice-packs to the laboratory, flash-frozen in liquid nitrogen within 24 h of sampling and stored at −80 °C until analyzed. For a complete description of the animals, please refer to [8,10]. Furthermore, unaffected Quarter Horses (*MYH1^E321G^*^−/−^; *n* = 2; one 1-year-old male and one 7-month-old female) with no history of neuromuscular disease consistent with equine MYHM were used as controls. Note that all the muscle samples were originally obtained for diagnostic purposes from client-owned horses. The sample submission form included a statement allowing the use of the tissue for research purposes. For the present study on these particular samples, we ethically obtained an IACUC AUF exemption approval.

### 2.2. Solutions

Relaxing and activating solutions contained 4 mM Mg-ATP, 1 mM free Mg^2+^, 20 mM imidazole, 7 mM EGTA, 14.5 mM creatine phosphate and KCl to adjust the ionic strength to 180 mM and pH to 7.0. Additionally, the concentrations of free Ca^2+^ ranged 10^−9.0^ M (relaxing solution, pCa 9.0) to 10^−4.5^ M (maximum activating solution, pCa 4.5). The rigor buffer contained 120 mM K acetate, 5 mM Mg acetate, 2.5 mM K2HPO4, 50 mM MOPS, and 2 mM DTT, with a pH of 6.8.

### 2.3. Muscle Preparation and Fibre Permeabilisation

Cryopreserved muscle samples were immersed in a membrane-permeabilising solution (relaxing solution containing glycerol; 50:50 *v*/*v*) for 24 h at −20 °C, after which they were transferred to 4 °C and bundles of approximately 50 muscle fibres were dissected free and then tied with surgical silk to glass capillary tubes at slightly stretched lengths. These bundles were kept in the membrane-permeabilising solution at 4 °C for an additional 24 h (to allow a proper skinning process). After these steps, bundles were stored at −20 °C for use up to one week [17].

### 2.4. Mant-ATP Chase Experiments

On the day of the experiments, bundles were transferred to the relaxing solution and single myofibres were isolated. Their ends were individually clamped to half-split copper meshes designed for electron microscopy (SPI G100 2010C-XA, width, 3 mm), which had been glued to glass slides (Academy, 26 × 76 mm, thickness 1.00–1.20 mm). Cover slips were then attached to the top (using double-sided tape) to create flow chambers (Menzel-Gläser, Braunschweig, Germany, 22 × 22 mm, thickness 0.13–0.16 mm) [18,19]. Subsequently, at 25 °C, for each muscle fibre, the sarcomere length was checked using the brightfield mode of a Zeiss Axio Scope A1 microscope (Jena, Germany). Fibres with a sarcomere length of ~2.50 µm were further subjected to the Mant-ATP chase protocol. Similar to previous studies [18,19], each fibre was first incubated for 5 min with a rigor buffer. A solution containing the rigor buffer with 250 μM Mant-ATP was then flushed and kept in the chamber for 5 min. At the end of this step, another solution made of the rigor buffer with 4 mM ATP was added with simultaneous acquisition of the Mant-ATP chase. For fluorescence acquisition, a Zeiss Axio Scope A1 microscope was used with a Plan-Apochromat 20x/0.8 objective and a Zeiss AxioCam ICm 1 camera. Frames were acquired every 5 s with a 20 ms acquisition/exposure time using a DAPI filter set, images were collected for 5 min. Three regions of each individual myofibre were sampled for fluorescence decay using the ROI manager in ImageJ as previously published [18,19]. The mean background fluorescence intensity was subtracted from the average of the fibre fluorescence intensity (for each image taken). Each time point was then normalized by the fluorescence intensity of the final Mant-ATP image before washout (T = 0). These data were then fit to an unconstrained double exponential decay using Sigmaplot:

Normalised Fluorescence = 1 − P1 (1 − exp^(−t/T1)^) − P2 (1 − exp^(−t/T2)^)

where P1 is the amplitude of the initial rapid decay approximating the ‘disordered-relaxed state’ (DRX) with T1 as the time constant for this decay. P2 is the slower second decay approximating the proportion of myosin heads in the ‘super-relaxed state’ (SRX) with its associated time constant T2. To obtain DRX and SRX values, P1 and P2 were adjusted for the level of non-specific binding found to be 40% in skeletal muscle [16].

### 2.5. Single Muscle Fibre Contractility

As for Mant-ATP experiments, individual myofibres were dissected in the relaxing solution. They were then individually attached between connectors leading to a force transducer (model 400A; Aurora Scientific, Aurora, ON, Canada) and a lever arm system (model 308B; Aurora Scientific Aurora, ON, Canada). Sarcomere length was set to ~2.50 µm and the temperature to 15 °C [20,21,22]. As the baths had glass bottoms and right angle prisms, fibre cross-sectional area (CSA) could be estimated from the width and depth, assuming an elliptical circumference. To determine the maximal and submaximal isometric force generating capacity, myofibres were sequentially bathed in activating buffers with increasing [Ca^2+^] termed pCa. Specific force corresponded to absolute force normalized to myofibre cross-sectional area. Submaximal force values were normalised to the maximal force and the obtained force-pCa curve were fit to the Hill equation (four-parameters). Ca^2+^ sensitivity or pCa_50_ was then obtained and corresponded to the pCa at which 50% of maximal force is reached. *n*H was also calculated and represented the steepness of the force-pCa curve, indicative of the degree of actin-myosin cross-bridge cooperativity [23].

### 2.6. Fibre Typing

As the mutation specifically affects the fast skeletal myosin heavy chain type IIx and as fibre typing for individual cells occurred after the mechanical experiments, we only kept the biophysical data from fibres expressing this specific isoform, i.e., the fast skeletal myosin heavy chain type IIx. Briefly, after the Mant-ATP chase experiments or contractile measurements, individual fibres were stained with an anti-myosin fast/type IIx antibody (IgM isoform, clone 6H1, dilution: 1:50, gift from Dr. Joseph Hoh—University of Sydney, Sydney, Australia) [24]. Myofibres were then washed in PBS/0.025% Tween-20, and incubated with a secondary antibody conjugated to Alexa 594 in a blocking buffer (from ThermoScientific, Waltham, MA, USA, dilution 1:500). After washing, muscle fibres were mounted in Fluoromount and images taken with a confocal microscope (Zeiss Axiovert 200, objective ×40) equipped with a CARV II confocal imager (BD Biosciences, Franklin Lakes, NJ, USA) [25]. The Supplementary Figure S1 depicts the pH 4.4 ATPase activity of fibres within the muscles of a control horse, a heterozygote, and an animal homozygous for the *MYH1* mutation.

### 2.7. Statistics

Data are presented as mean ± standard deviations. The statistical analysis was performed using SPSS Statistics 23.0 (IBM, Armonk, NY, USA) and significance was set to *p* < 0.05. The one-way ANOVA statistical test was applied, and in cases where the data did not meet the criteria of normality, the Kruskal–Wallis one-way ANOVA test was performed. Note that data are available on request.

## 3. Results

All the results presented below focused on myofibres expressing the fast skeletal myosin heavy chain type IIx where the subtle amino acid substitution occurs.

### 3.1. The Proportion of Myosin Heads in the Super-Relaxed State Is Preserved in the Presence of MYH1^E321G^

**Figure 1:**
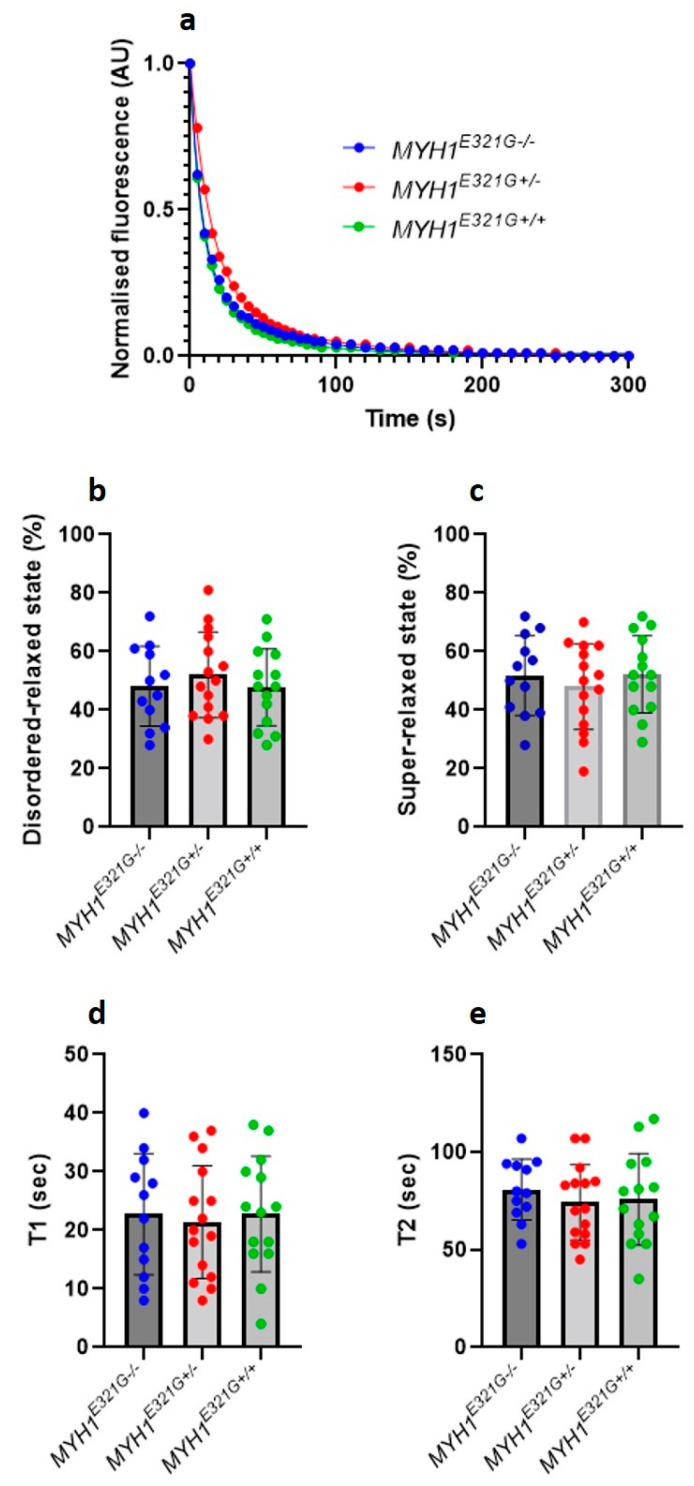
Myosin relaxed states in individual muscle fibres. (**a**) Typical Mant-ATP chase experimental data showing exponential decays for muscle fibres isolated from control horses (*MYH1^E321G−/−^*) and Quarter Horses homozygous (*MYH1^E321G+/+^*) or heterozygous for the mutation (*MYH1^E321G+/−^*). The proportion of myosin molecules in the super-relaxed state or disordered relaxed states as well as their ATP turnover lifetime were estimated from the equation presented in the methods section. The amount of myosin molecules in the super-relaxed (**b**) and disordered relaxed states (**c**) did not differ between the groups. Similar observations were made for ATP turnover of myosin in the super-relaxed ((**d**), T1) or disordered relaxed states ((**e**), T2). Dots are individual muscle fibre data. Means and standard deviations also appear on histograms.

Super-relaxed myosin molecules have very low ATPase activity in comparison to its other conformational states (e.g., disordered-relaxed state) [16]. By using this assumption and the Mant-ATP chase protocol [16], in the present study, we assessed the proportion of myosin heads in the super-relaxed or disordered-relaxed states and did not observe any significant differences between muscle fibres from *MYH1^E321G+/+^* (*n* = 14), *MYH1^E321G+/−^* (*n* = 15) and *MYH1^E321G−/−^* (*n* = 12) horses (Figure 1a–c). Similarly, the lifetimes of ATP turnover of myosin in the super-relaxed or disordered-relaxed states were unchanged between the groups (Figure 1d,e).

### 3.2. MYH1^E321G^ Is Associated with an Increased Muscle Fibre Force Generating Capacity and Ca^2+^ Sensitivity

**Figure 2:**
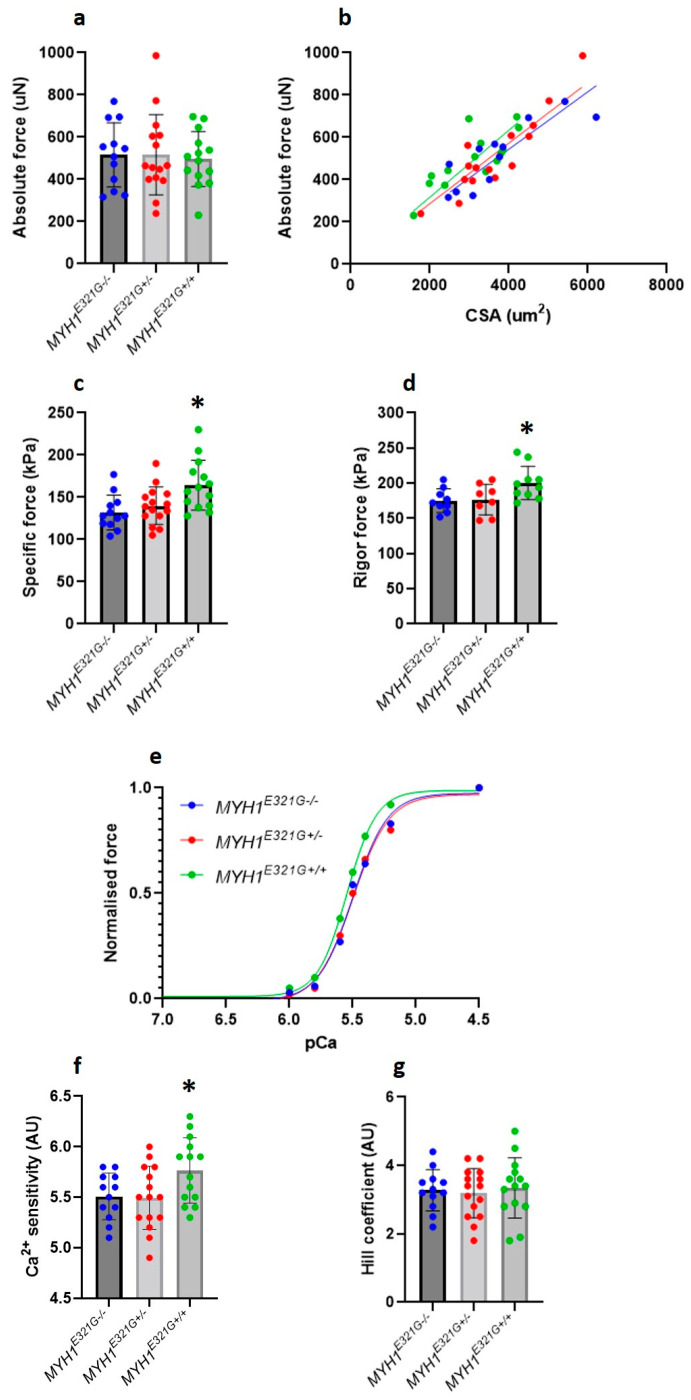
Contractile properties of isolated myofibres. (**a**) Absolute force. (**b**) Absolute force–myofibres cross-sectional area (CSA) relationship. The maximal (**c**) and rigor (**d**) (specific) force production was higher in *MYH1^E321G+/+^* when compared to *MYH1^E321G+/−^* and *MYH1^E321G−/−^* animals. (**e**) Typical data depicting the force generating at various Ca^2+^ concentrations (pCa) for myofibres from *MYH1^E321G+/+^*, *MYH1^E321G+/−^* and *MYH1^E321G−/−^* animals. (**f**) The Ca^2+^ sensitivity (pCa_50_) was greater in *MYH1^E321G+/+^* when compared to *MYH1^E321G+/−^* and *MYH1^E321G−/−^* horses. (**g**) The Hill coefficient or cooperativity (*n*H) was not different between the groups. Dots are individual muscle fibre data. Means and standard deviations also appear on histograms. Stars represent a significant difference with *MYH1^E321G−/−^* horses (* *p* < 0.05).

Despite a maintained super-relaxed state in the presence of E321G, we further experimentally evaluated whether the mutation would modify myosin active states. For that, we analysed the force-generating capacity of individual membrane-permeabilised muscle fibres at different [Ca^2+^] (Figure 2a,b). The maximal (specific) force produced and Ca^2+^ sensitivity (pCa_50_) were significantly greater in muscle fibres from *MYH1^E321G+/+^* (*n* = 14), when compared with *MYH1^E321G+/−^* (*n* = 15) and *MYH1^E321G−/−^* (*n* = 12) horses (Figure 2c,e,f). However, cooperativity (nH) was not affected by the mutation (Figure 2g). To get further insights into the mechanisms underlying the cellular hyper-contractile phenotype with E321G, we further evaluated the maximal (specific) force for a sub-set of myofibres under rigor conditions, where all myosin heads are attached due to the absence of ATP and a very slow dissociation rate. Rigor (specific) force was significantly higher in myofibres from *MYH1^E321G+/+^* (*n* = 10), when compared with *MYH1^E321G+/−^* (*n* = 8) and *MYH1^E321G−/−^* (*n* = 9) horses (Figure 2d).

## 4. Discussion

Our findings indicate that, contrary to our initial hypothesis, the E321G amino acid substitution in the head domain of myosin heavy chain IIx, does not alter the super-relaxed conformation of the motor proteins and its ATP consumption within thick filaments, sarcomeres and myofibres. Whilst nearby mutations (R369Q, E374V, A381D and Y386C) related to cardiomyopathies are all associated with a disruption of the myosin interacting-heads motif [15], it remains unclear why E321G does not interfere with the conformations of relaxed heads. This specificity is unlikely to be related to the biochemical properties of the mutation as the E to G replacement induces a similar effect (from a negatively charged residue to a non-polar amino acid) as the E to V substitution. On the other hand, one potential explanation may lie in the exact location of the mutation. E321G is in the I-loop whereas R369Q, E374V, A381D and Y386C are in the C-loop [8,15]. Besides no change in the super-relaxed state, our results indicate that the E321G residue replacement modifies the active binding of myosin molecules to actin filaments (as attested by the enhanced force productions at various Ca^2+^ concentrations). This is in line with the neighbouring mutations mentioned above [15] and this is expected, as the subtle defect is located in the motor region of myosin heavy chain IIx. An actin-binding alteration can be due to either a change in the number of strongly attached myosin cross-bridges and/or a modification of the force per individual actomyosin interaction. To distinguish between these two potential mechanisms, here we measured rigor force. This latter was greater in the presence of E321G, indicating that the mutation may lead to an increase in the force developed by individual cross-bridges rather than a greater number of myosin heads strongly bound to actin filaments.

The exact biological consequences of our findings are unclear; however, a large number of mutations linked to hypertrophic cardiomyopathy suggest that molecular and cellular hyper-contractility has mechanical effects among the tightly interconnected neighbouring sarcomeres and myofibres. Indeed, unusually high forces on these structures may induce distortions or ruptures within adjacent units [26]. This could be one explanation for the initial stiffness, firm contracted muscles and non-exertional rhabdomyolysis experienced by MYHM horses [10,11,27]. Skeletal muscle proteins like myosin appear to be partially sequestered from the immune system with very little expression of major histocompatibility complex class I and II (MHC) antigens on the sarcolemma in healthy horses [12]. Horses with active immune-mediated myositis, in contrast, have MHCI and II expression in a certain number of myofibres [12]. One plausible cause for the immune-mediated myositis in horses with the *MYH1^E321G^* mutation could then be exposure of the unique myosin heavy chain to the immune system following excessive contraction and cellular damage, which then triggers the innate immune-response altering reactivity to self [8]. Exposure to respiratory or gastrointestinal infections and vaccinations are other activators of the immune system that can induce muscle atrophy and immune-mediated myositis in 40–50% of horses with clinical disease, potentially due to epitope sharing [11,27].

Although *MYH1^E321G+/+^* homozygotes are rare in random sampling of Quarter Horses (< 1%) relative to heterozygotes (*MYH1^E321G+/−^*), they appear to comprise the largest proportion of horses with immune-mediated myositis (68%) and rhabdomyolysis (75%) [8,9]. Many of the heterozygous horses do not develop MYHM [8]. In line with this, our examination of IIx muscle fibres from *MYH1^E321G+/−^* animals did not reveal any significant effects on myofibre contractility. Note this could be a sample size issue, as unfortunately, our study was limited by the number and size of well-preserved muscle biopsies available. It is possible that a portion of the fibres examined from heterozygotes did not express the *MYH1^E321G^* mutant or expressed variable amounts (as previously shown in the presence of human *MYH7^R723G^* and *MYH7^R200V^* mutants [28]). This would limit our ability to detect a cellular functional difference in this particular genotype. The number of horses evaluated in the present study was limited and should be expanded in the future. It is of note, however that specific force was previously assessed by our laboratory in a study of Warmblood horses (eight horses, 27 fibres) with values (140.2 ± 43.7 kPa) that were very similar to control horses in the present study [29]. Determining the proportion of mutated type IIx skeletal myosin heavy chains may be crucial in the development (or not) of MYHM and this should be addressed in a future study. Additionally, in future experiments, we should assess how our findings relate to newly reported *MYH1*-related human rhabdomyolysis cases [30].

## 5. Conclusions

To conclude, our study revealed that E321G disrupts myosin active functioning but not its relaxed conformations. This hyper-contractile molecular and cellular phenotype is highly likely to contribute to the development of equine MYHM and could then be targeted in future therapeutic interventions.

## Data Availability

Data are available on request.

## Supplementary Materials

The following are available online at https://www.mdpi.com/article/10.3390/cells10123428/s1, Figure S1: Myosin ATPase activity.
