# Supplementary material for: Myofibre Hyper-Contractility in Horses Expressing the Myosin Heavy Chain Myopathy Mutation, MYH1E321G
Source: Cells. 2021 Dec 6;10(12):3428. doi: 10.3390/cells10123428 (PMC8699922; doi:10.3390/cells10123428)
Supplement: Supplementary file 1 [file cells-10-03428-s001.zip › cells-1460800-supplementary.pdf]

Supplementary Figure

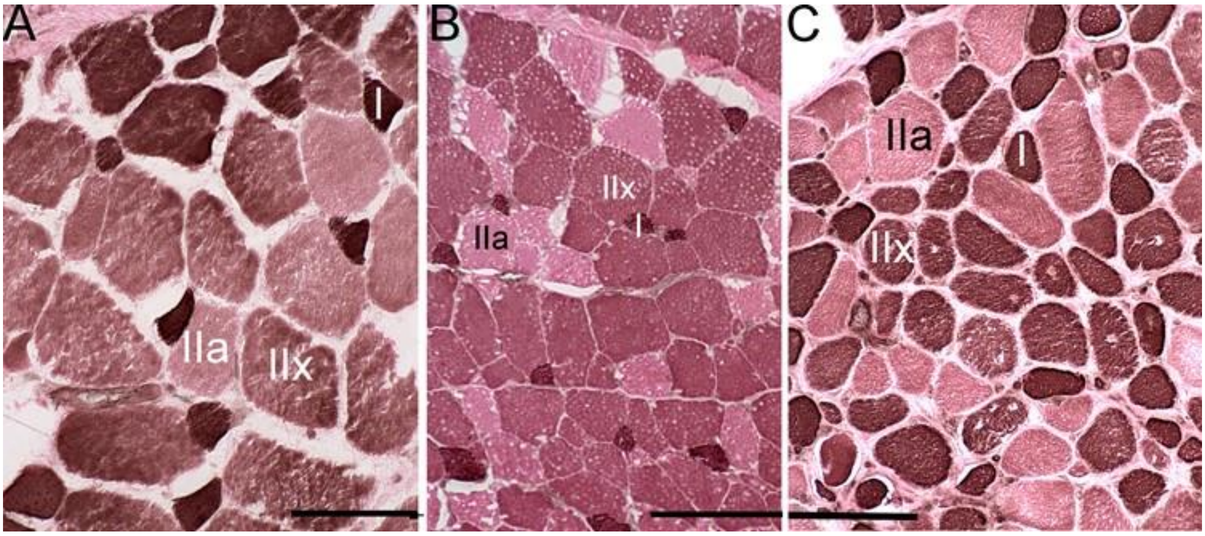

Figure S1. Myosin ATPase activity. Myosin ATPase activity (pre-incubation pH 4.4, eosin counterstain) of a cross-section of muscle from a control Quarter Horse (**A**), a heterozygote (**B**) and a horse homozygous for the *MYH1*^{E321G} mutation (**C**). All horses were between 7 months and 1 year of age. Notable IIx fibre size variation was present in the horse homozygote for *MYH1*^{E321G}.
